# Reassessing the econometric measurement of inequality and poverty: toward a cost-of-living approach

**DOI:** 10.1057/s41599-023-01738-3

**Published:** 2023-05-11

**Authors:** Anson Au

**Affiliations:** Department of Applied Social Sciences, The Hong Kong Polytechnic University, 11 Yuk Choi Road, Hung Hom, Kowloon, Hong Kong

**Keywords:** Sociology, Economics

## Abstract

The econometric measurement of inequality and poverty in advanced capitalist economies has been preoccupied with aggregate measures of relative deprivation, namely, the Gini Index and a relative poverty rate, both of which are based on economic distances from the population median. Using the case of Hong Kong, this article demonstrates the limitations of relative measures: the Gini Index masks social mobility and the relative poverty line understates actual poverty. This article argues instead for a cost-of-living approach to measure poverty, where the poverty line is defined as the cost of essential goods and services. A cost-of-living approach produces a poverty line of HK$28,815 and attendant poverty rate of 44.47% in 2020, nearly double the poverty line of HK$13,450 and poverty rate of 23.6% according to the conventional relative measure of the poverty line set to 50% of median household income—capturing a shortfall of 551,400 poor households that have been overlooked by relative measures.

## Introduction

The measurement of inequality and poverty in a society has direct implications for household wellbeing by determining the threshold for government program payouts like welfare and the size of payouts required, but the methodology for which these measures are determined has been contested since the 1970s (Atkinson, 1998; Gordon, 1972; Sen, 1973, 1982).

The destabilization of the income distribution during this period led to a universal move to adopt the Gini Index as a gold standard measure for assessing inequality, which is a scaled measure of the sum of individual income differences from the median income in a population. This relative measure thus aims to depict inequality with an aggregate level distribution (Antonelli and Rehbein, 2018; DiPrete, 2007; Myles, 2003; Osberg, 2017).

Similar debates have emerged over the measurement of poverty, with the most recent consensus concluding that absolute measures, such as the World Bank’s (2021a, b) famous US$1 to US$2 income per day threshold, are claimed to be better for developing nations by capturing absolute levels of material purchases deemed necessary for survival (Sen, 1982). However, absolute measures fail in accounting for the economic and social context within which needs arise. Such is the issue in the case of China, where, a recent study on the *dibao*, China’s welfare, identify that the rural *dibao* program excluded 87% of the poor and 82% of the non-poor in 2013 (Kakwani et al., 2019). This problem was sourced to the methodology for deciding poverty, which originally began as an attempt to determine absolute levels of needs, but eventually percolated into decentralized decision-making by village administrations that struggled to determine these levels (Kakwani et al., 2019, p.7).

This issue surfaces in the understatement of poverty rates by official measures of poverty that rely on an arbitrary US$1 to US$2 a day poverty threshold (World Bank, 2021a, b), despite successes in reducing poverty (Chen, 2008; Zhang et al., 2014). Turning their attention to the current *dibao*, Walker and Yang (2021) find that if China adopted the global standard for poverty that is based on relative measures—classifying one is poor if they are making less than 40 to 60% of the median income in the entire population—the poverty rate would shoot up to 8% to over 20%. Similarly, Wan et al. (2021) find that the same fast economic growth that explains declines in absolute poverty in China (based on the $1 to $2 a day poverty threshold) is also what explains a rising relative poverty rate.

Indeed, the global consensus for the measurement of poverty is gradually shifting to the adoption of relative measures akin to the consensus for Gini Index as a relative measure of inequality. The poverty threshold according to relative measurements of poverty, such as household incomes that fall below a certain percentage (40 to 60%) of the median income, are now widely considered to capture changes in household needs over time, especially in the case of developed economies or countries like those in the Asia-Pacific that have moved from low-income to middle-income status by GDP per capita. Such measures, according to Brady (2003, 2019), are argued to capture needs because needs themselves are relative and contextualized within a given society to begin with, highly dependent on reference groups that individuals have within a society.

Recent political progressivism has sedimented this fascination with relative measurements, couched in slogans with popular political appeal that binarizes economic disenfranchisement in classes, like the “99%” poised against the “1%” in social movements such as Occupy Wall Street and newly emerging progressive agendas in the U.S. and China (Calhoun, 2013; Kakwani et al., 2022; Stroh, 2020; Van Gelder, 2011). Indeed, contemporary measurements of inequality by social scientists and the World Bank consistently measure it on an aggregate level by using the Gini Index (Liao, 2021; Oishi et al., 2011; Pickett and Wilkinson, 2015; Zagorski et al., 2014).

In conversation with this literature, this article contributes to the methodological study of inequality and poverty by demonstrating the limitations of relative measures, such as to fail to capture social mobility and understating actual poverty—and arguing instead for a cost-of-living approach to measurement. Qualitative and quantitative studies of the trauma of poverty demonstrate the myriad challenges faced exclusively by people who do not have enough to survive: including predisposition to mental health issues and disabilities from a lifetime of homelessness and eviction, cultural norms of discrimination, stigma, and shame on the basis of their class in access to social services, housing, education, and work, as well as exposure to illicit drug and violent activities as one of the few means of economic subsistence available (Beddoe and Keddell, 2016; Contreras, 2013, 2017; Desmond, 2012, 2016; Desmond and Kimbro, 2015; Desmond and Western, 2018; Desmond and Wilmers, 2019; Hansen et al., 2014; Sánchez-Jankowski, 2008, p. 48; Shildrick, 2018).

Apart from its consequential or theoretical significance, a cost-of-living approach better accounts for inelasticity of certain material needs. Relative measures of poverty like 50% of the median income have been argued to capture the dependence of needs on reference groups in different social contexts, for what individuals are willing to spend or what they purchase may be dependent on what their communities or neighborhoods purchase (Brady, 2019), but this assumption erroneously overstates income and price elasticity of all needs. According to the National Bureau of Economic Research (2016), for instance, housing demand is actually income and price inelastic (Albouy et al., 2016). Rising relative rents, moreover, produce increases in real income inequality that are missed by relative measures of poverty and inequality. This finds resonance with Piketty’s (2014) observation that the value of land occupies a greater proportion of the economy because of this very same inelasticity in the demand for land, such that the price of housing has risen almost 40% more than other goods since 1970 in developed economies (Albouy et al., 2016).

This article addresses this limitation by using the case of Hong Kong to demonstrate the merits of a cost-of-living approach to measuring inequality and poverty over relative measures. Hong Kong is an urban city with a population of around 7 million people and an average GDP per capita is 49,800 USD as of 2021. Of its 3.67 million employed workers, according to the latest Census (2021a), 1.102 million work in public administration, social and personal services (30.03% of the workforce), 862.4 million in financing, insurance, real estate, professional, and business services (23.5% of the workforce), 430.2 million in transportation, storage, information and communications (11.72% of the workforce), 516.4 million in retail, accommodation, and food services (14.06% of the workforce), 316.4 million in import/export trade and wholesale (8.62% of the workforce), 325.8 million in construction (8.88% of the workforce), and 94.3 million in manufacturing (2.57% of the workforce). Thus, as an advanced capitalist economy with an entirely service-based workforce, Hong Kong is an apposite case for comparing relative versus cost-of-living measurements of inequality and poverty in advanced capitalist economies at large, especially those in the Asia-Pacific where empirical evidence has been limited (Brady and Burton, 2016). Through a cost-of-living approach, the Gini Index and relative poverty are demonstrated to be insufficient in understanding within-population shifts in economic wellbeing and to understate the poverty rate among households.

## Problems with relative measures of inequality and poverty

This section identifies the problems with relative measures of inequality and poverty, namely, the Gini Index and relative benchmarks of poverty as a proportion of the median income.

The Gini Index is one of the widest used measures of economic inequality, most of all in sociology. Devised by Gini in 1912, the Gini Index expresses a concentration ratio equal to the average distance between *n* quantities divided by double the arithmetic mean of a generic income distribution *X*. In a population of *N* individuals, *i* = *1,2…n, n* ∈ *N, n* ≥ *3* with an income distribution of *X* = (*x*_1_, *x*_2_… *x*_*i*_…*x*_*n*_) where *X* ∈ *R* and *x*_1_ ≤ *x*_2_ ≤ … ≤ *x*_*n*_, an arithmetic mean of *μ*_*X*_, and *x*_*M*_ is the individual with the median income:

1 $$G\left( X \right) = \mathop {\sum}\nolimits_{i = 1}^n {\frac{{2\left( {i - M} \right)\left( {x_i - x_M} \right)}}{{n^2\mu _X}}}$$

*G(X)* is thus a scaled measure of all possible pairwise differences or individual diversity in income, through which it satisfies the six desirable properties of an inequality measure: continuity, additivity, linear homogeneity, translation invariance, symmetry, and anonymity (Ceriani and Verme, 2015). Configurations have since been made to the Gini Index, of which Ceriani and Verme (2012) identify thirteen. Such forms in which the Gini Index is expressed (and the individual functions underlying the different Ginis) consist of distances from the median, geometric mean, covariance, etc. (Anand, 1983; Gini, 1914; Sen, 1973). Distributions computed using Gini Indices, however, fundamentally rely on individual contributions based on endogenous values that conceptualize the distribution squarely as expressions of relative deprivation (Yitzhaki, 1979).

Attempts to broaden the application of the Gini Index have also yielded alternative configurations that decompose it at subgroup levels, such as when a population is classified into *K* number of groups for *k* = 1 to *K*, where *G(X)* is based on (a) the income difference between individual *i* in group *k* and other individuals *j* in a different group *h*, (b) the income difference between individual *i* and other individuals *j* in the same group *k*, such that *n*_*h*_ is the size of the group *h* and *n*_*k*_ is the size of group *k*, *h* *≠* *k*:

2 $$G\left( X \right) = \frac{{\mathop {\sum}\nolimits_{h = 1}^K {\mathop {\sum}\nolimits_{j = 1}^{n_h} {\left| {x_{ik} - x_{jh}} \right|} } }}{{2n^2\mu _X}} + \frac{{\mathop {\sum}\nolimits_{j = 1}^{n_k} {\left| {x_{ik} - x_{jk}} \right|} }}{{2n^2\mu _X}}$$

Nonetheless, these configurations converge on the same prototypical model of relative deprivation, whereby individual contributions to inequality are based on individual *i* diversity from other individuals *j* richer or poorer. As a result, this model and its configurations are ultimately poorly sensitive to changes in income and to whether pairwise differences are positive or negative (Alvaredo and Piketty, 2014; Atkinson et al., 2011; Ceriani and Vermea, 2015; Cowell and Ebert, 2004). The theoretical and empirical limitations of the Gini Index and relative deprivation thus rise to the fore.

Relative deprivation fails to capture material deprivation, or the ability to afford the cost of living, typically equated with the poverty line below which individuals within a population *X* are said to be living in poverty *P* in a given social unit often defined at the levels of cities, states or provinces, and nations.

Consider that in the Asia-Pacific (Table 1), the movement of the Gini Index is not in pace with the poverty rate: the Philippines exhibits a higher poverty rate than Hong Kong despite its markedly lower Gini Index.

**Table 1:** Gini Indices and poverty rates of Hong Kong, India, and Philippines.

	Hong Kong	India	Philippines
*G(X)*	0.54	0.357	0.423
*P(X)*	23.6%	11.3%	23.7%

Sources: Census and Statistics Department of Hong Kong (2021b), Philippine Statistics Authority (2021), The World Bank (2021a, b).

The discrepancy between the Gini Index and the poverty rate is because changes to income—upward or downward—yield comparable distributions and, since the Gini measures scaled differences, they resultantly pass without detection in the Gini Index. Yet, it is in these changes that we glimpse powerful antecedents of poverty and social mobility in general. A Gini Index neglects, for instance, to identify broader patterns of material deprivation among households across time that would otherwise allow for subsequent analysis of their causes among externalities, such as the effects of inflation on lower-income households.

**Fig. 1:**
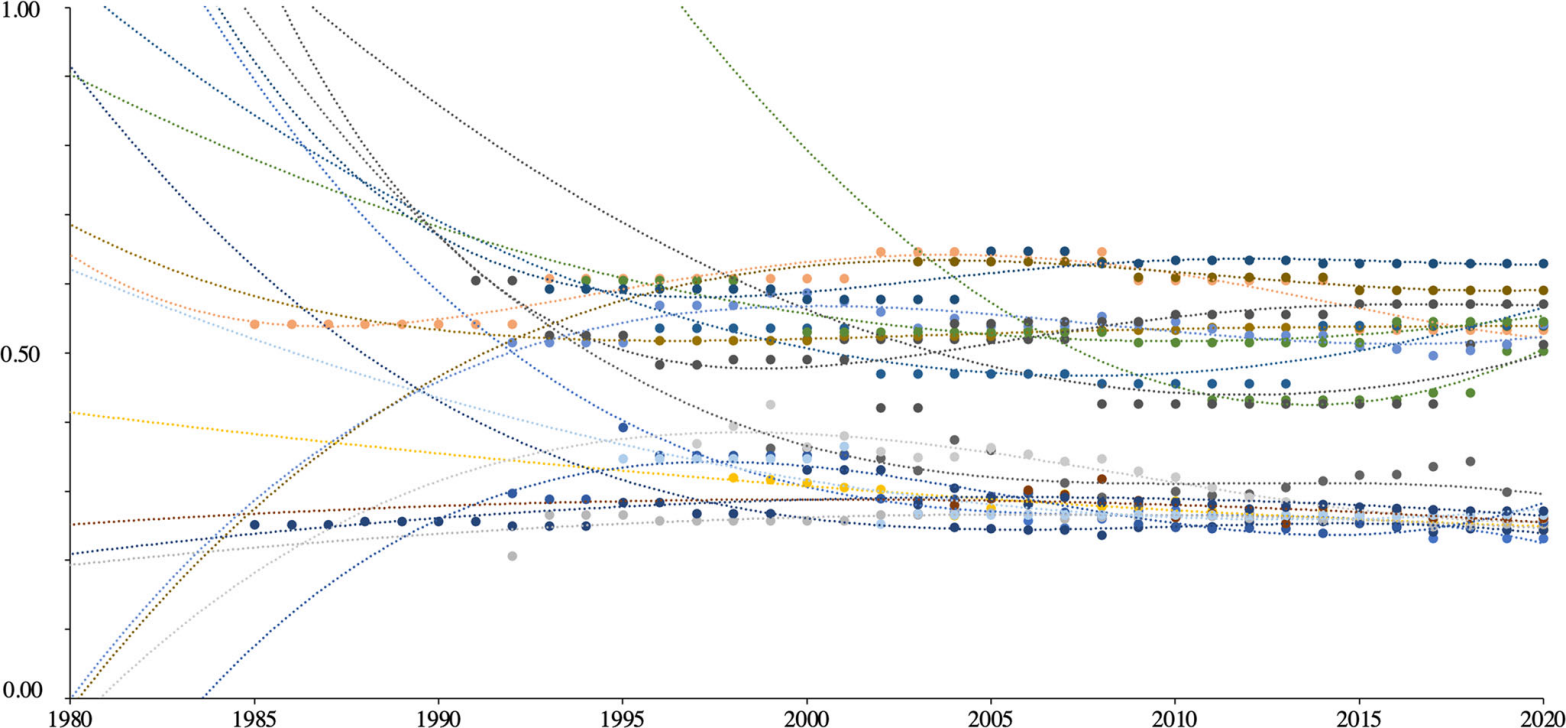
Gini Indices of the top ten societies with the highest and lowest Gini indices. Source: Author’s calculations using data from the World Bank (2021a).

It follows that a Gini Index will fail to detect underlying shifts in economic wellbeing on upper and lower bounds so long as both ends experience changes that are relatively symmetrical. It also follows that derivations of the Gini Index over time will likely exhibit horizontal asymptotic tendencies once a society reaches a certain level of inequality. An examination of the Gini Indices of the ten societies with the highest Ginis and ten with the lowest illustrates this behavior (Fig. 1).

For societies on the lower bounds, as time *t* approaches infinity, Gini Index *G(X)* approaches 0.2. For societies on the upper bounds, there exist several asymptotes as time *t* approaches infinity, but these are consistently placed at a Gini Index *G(X)* of about 0.5 and 0.6. The chief problem identified in these observations is that the Gini Index tends to stagnate, which erroneously suggests stalls in inequality, but for reasons endogenous to the way it is derived, rather than due to underlying shifts in economic means or pressures—casting doubt on the Gini’s efficacy as a meaningful measurement of economic wellbeing (and underlying expression of relative deprivation) over time.

The poverty rate is commonly calculated by the Organisation for Economic Co-operation and Development (2016) using 50% of median household income (other configurations have since been developed to push the rate to 30% in deciding the line of poverty) as a poverty line, roughly approximated as the following where *z* indicates individual income and *u* is median income in a population *X*:

3 $$P\left( X \right) = \frac{{{\sum} {\left[ {X_z < \left( {0.5 \times u} \right)} \right]} }}{X}$$

## Measuring poverty based on cost-of-living

The present article articulates a cost-of-living approach to reassess the poverty rate, rather than economic distances in income endogenous to a population. The poverty rates reported in Table 1 was based on (3). Yet, this benchmark is ultimately arbitrary and premised on the idea of income differences within the same population that resembles once more relative deprivation. As such, the poverty line based on (3) may fluctuate drastically and *decrease* in economic downturns because of a lower median income *despite* greater material deprivation as more people cannot afford basic goods and services.

The latest COVID-19 pandemic is a useful heuristic. In 2020, most economies worldwide suffered drops in Gross Domestic Product (and per capita) as travel and cross-border services shrivelled up, trade wound down, lockdowns sent cities and small and medium-sized enterprises shuttering down—and as a result, unemployment rates rose drastically. Poverty rate (3) thus *understates* the amount of actual material deprivation in a given society.

By contrast, benchmarking the poverty rate to cost of living captures far more meaningfully whether people can afford to survive and under what living conditions, particularly when income elasticities are unitary. Cost-of-living *L* in a population *X* can thus be calculated in two steps, where *F* is an index for changes in real prices of goods and services, *C*_*g*_ is the cost of goods and *Q*_*g*_ is the quantity of goods at time *t*, *t* = 0, 1…*a*, *a* ∈ *N*, *n* ≥ 3 and *t* = 0 is the base period:

4 $$F = \sqrt {\frac{{{\sum} {\left( {C_{g,t = a} \times Q_{g,t = 0}} \right)} }}{{{\sum} {\left( {C_{g,t = 0} \times Q_{g,t = 0}} \right)} }} \times \frac{{{\sum} {\left( {C_{g,t = a} \times Q_{g,t = a}} \right)} }}{{{\sum} {\left( {C_{g,t = 0} \times Q_{g,t = a}} \right)} }} \times 10,000}$$

Then,

5 $$L\left( X \right) = C_{g,t = 0}Q_{i,g,t = a}\left( {\frac{{F_{t = a}}}{{F_{t = 0}}}} \right)$$

(4) is effectively the Fisher Price Index that captures changes in real prices of goods and services, which is presently integrated into (6) a revised formula for the poverty rate *P* of a population *X* (or proportion of population *X*) living below (5) the cost of living *L(X)* defined as the necessary amount *Q*_*g*_ of goods *C*_*g*_ for an individual *i* to consume in year *t* = *a* pro-rated to the base period *t* = 0, where *I* is an indicator function such that individuals are counted as poor (*I* = 1) if individual income *y*_*i*_ during the same period (between base period *t* = 0 and *t* = a) is below cost of living *L(X)* and individuals are counted as non-poor (*I* = 0) if *y*_*i*_ is above *L(X)*.

6 $$P\left( X \right) = \frac{1}{X}\mathop {\sum}\nolimits_{i = 1}^X {I\left[ {y_i \le L\left( X \right)} \right]}$$

Equation (6) offers a methodologically flexible measure of material deprivation by leaving room for theoretical assumptions about the scope of study and population needs, namely interval *t*, the base period at which *t* = 0, and the amount of goods *Q*_*g*_ needed to be consumed by individuals. This flexibility ultimately permits its application across societies, accounting for, rather than obscuring, differences by geographical contexts (Brady, 2003).

## Re-examining inequality and poverty in Hong Kong

**Fig. 2:**
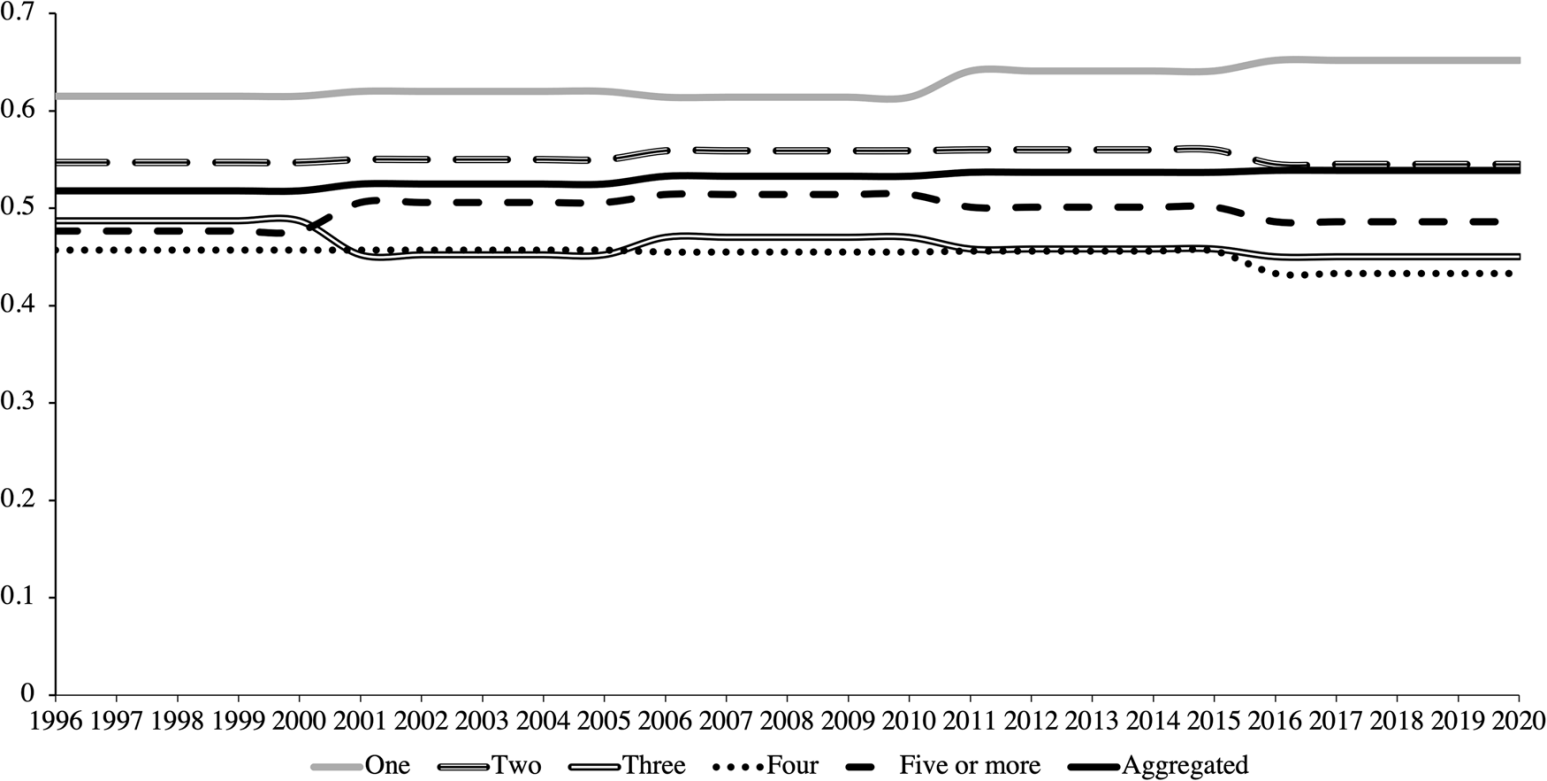
Gini Indices decomposed by groups according to household size over time. Source: Author’s calculations using data from the Legislative Council of Hong Kong (2001), Census and Statistics Department of Hong Kong (2021b), and Social Indicators of Hong Kong (2022).

How is the economic wellbeing of Hong Kong residents? Let us consider the region’s household financial data over the past twenty-five years from 1996 to 2020. An aggregated Gini Index calculated using Eq. (1) produces a value within an inflexible range of about 0.52 to 0.54, effectively holding constant for the entire period (Fig. 2). Taken at face value, this means that inequality has not worsened for twenty-five years, bad as it may be. This is consistent with global trends of the Gini Index that show significant stagnation over time (Fig. 1).

Applying Eq. (2) to decompose the Gini Index by groups, defined as households of different size, produces a marginal improvement (Fig. 2). All values moved beyond one standard deviation from the mean during this period, but the most significant moves in absolute terms were by households of one with a rise of 6.02% from a Gini of 0.615 to 0.652, households of three with a decline of 7.60% from a Gini of 0.487 to 0.45, and households of four with a decline of 5.25% from a Gini of 0.457 to 0.433, with the rest largely staying stagnant. Inequality appears to have gotten worse for households of one only, with the rest improving or staying within the same Gini brackets.

Though some stratification by household size is visible at a group level with Eq. (2), the Gini Index does not permit the visualization of the size or scope of such stratification or, relatedly, social mobility, which may be conflated with demographic patterns like changes in household sizes over time in general. Indeed, any attempt to observe social mobility out or into poverty is complicated by demographic shifts in household size.

**Fig. 3:**
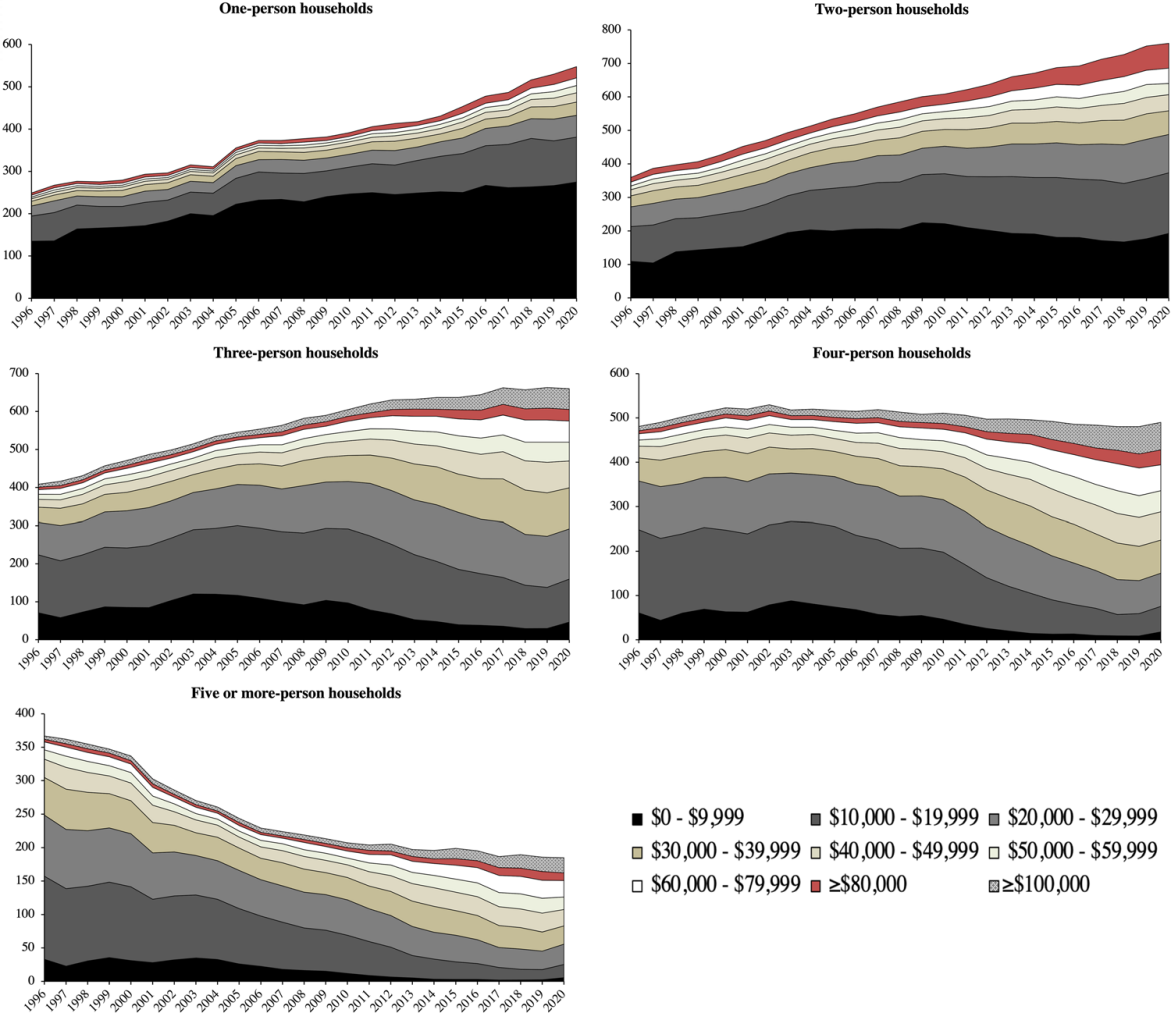
Number of households at various levels of household income in thousands, differentiated by sizes of household (1996–2020). Sources: Author’s calculations based on data from the Census and Statistics Department of Hong Kong (2021b).

Decomposing household income by household size in absolute numbers of individuals rather than as relative proportions in Fig. 3, we observe nuances masked by the Gini Index. Households of one exhibited the greatest jump in inequality in Fig. 2, but Fig. 3 shows us that this inequality is understated: we observe the greatest demographic growth in households of one (and two), and most of this growth was sequestered in the lowest income bracket (those earning HK$0 to HK$9,999 per month). More than a portrait of inequality, this sensitizes us to a growing issue of *poverty*.

Household sizes of two, which showed the greatest inertia in the Gini Index calculated with Eq. (2) in Fig. 2, show growing inequality in Fig. 3, as the absolute numbers of people in the highest (those making HK$80,000 or more per month) and lowest income brackets (HK$0 to HK$9,999 per month) have grown the most. This sensitizes us to the problem of the Gini Index as a scaled measure, where, even using configurations at a group level with Eq. (2), it is insensitive to social mobility and changes in income when upper and lower bounds exhibit symmetry in scalar changes.

Household sizes of three and four showed declines in inequality in Fig. 2, but we observe in Fig. 2 that this owes to a steep decline in the number of households with the lowest income (HK$0 to HK$9,999 per month). In a similar vein, households of five or more persons were shown to stagnate in Fig. 2, but this masks the greatest demographic collapse out of all household sizes. Examining economic means expressed in absolute numbers in Fig. 3 rather than relative scales in Fig. 3 thus warns us not to overinterpret declines or stagnation in the Gini as the equitable redistribution of economic means, but rather, as a trend in social mobility rooted in a dislocation between economic pressures and individual economic means. Reconciling these declines in the Gini Index with the large growth in the number of households of one and two persons, it is more plausible that a greater number of people are being siphoned into lower incomes over time and that those earning the lowest income have not been able to afford to get married or have children (and co-reside with them). This is also reflected in the general inverted relationship between household size and number of households over time in all households in Fig. 3, signaling aggregate shifts in the general population from larger household sizes to smaller ones.

Underwritten in the limitations of the Gini Index (and adherent configurations, Eqs. (1) and (2)) as its measure is the identification of the growing problem of—and need to examine—material deprivation. Adopting a cost-of-living approach thus leads us to evaluate the poverty rate using the standard measure of poverty as a proportion of median income, Eq. (3), and on the basis of real cost-of-living, Eq. (6).

**Fig. 4:**
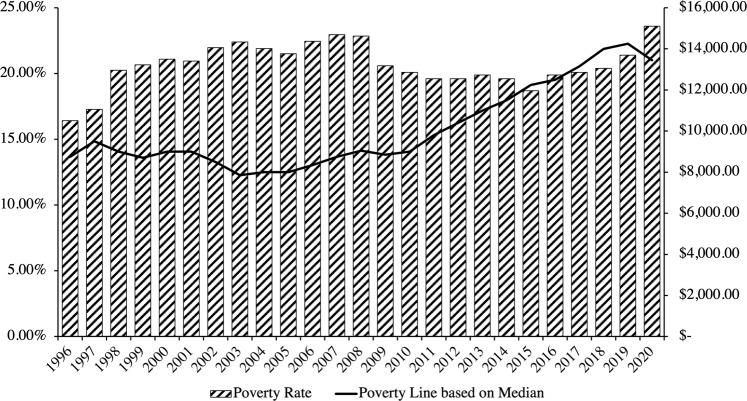
Poverty rate and poverty line of Hong Kong based on standard configurations of Eq. (3). Source: Author’s calculations based on data from the Census and Statistics Department of Hong Kong (2021b) and the Legislative Council of Hong Kong (2001).

Using Eq. (3), the poverty line and attendant poverty rate in Hong Kong are estimated in Fig. 4. Based on government data, it is observed that the Hong Kong government has historically defined the poverty line to be 50% of median (monthly) household income for a household size equivalent of about 2.5 persons (*Q*_*g*_ = 2.5). Equation (3) has been configured accordingly. The poverty line is thus HK$13,450 in 2020 compared to HK$8,750 in 1996.

Figure 4 reveals that the poverty rate is alarmingly high, hovering consistently around 20% for the better part of the past thirty years. Even still, however, we observe from Fig. 4 that the poverty rate calculated using Eq. (3) understates material deprivation because of its reliance on economic distances from the median as a relative—not absolute—determination of the poverty line.

To illustrate, let us consider four periods of economic crisis in Hong Kong: the 1998–1999 Asian Financial Crisis, the 2001–2003 recession, the 2008–2009 Financial Crisis, and the 2020 COVID-19 pandemic. These four crises inflicted the greatest hits to households’ economic means measured in losses of income and steep rises in the unemployment rate, coupled with rising Consumer Price Index (CPI) inflation (Table 2). It deserves noting that the unemployment rates for all four crises are higher than the mean, with three of the four rising to one standard deviation above the mean.

**Table 2:** Comparisons of changes in economic means and costs during economic crises.

	Economic means		Economic costs	Lag
Economic crisis	Change in median household income	Unemployment rate	Inflation	Inflation—change in median household income
1998–1999 Asian Financial Crisis	−8.60%	5.42%	−1.20%	7.40%
2001–2003 Recession	−13.2%	6.74%	−7.20%	5.00%
2008–2009 Financial Crisis	1.22%	4.79%	4.80%	3.58%
2020 COVID-19 Pandemic	−5.61%	5.80%	0.30%	5.91%

Source: Author’s calculations based on data from the World Bank (2021b) and the Census and Statistics Department of Hong Kong (2021b).

Since the poverty line is pegged to household median income, changes in the former are equivalent to those in the latter. From the comparisons between means and costs, we may further estimate whether a lag exists in the ability of the poverty line to capture all changes in cost-of-living for households. A positive difference between inflation and change in median household income will indicate a lag, where adjustments to the poverty line, equivalent to changes in median household income, fail to keep pace with changes in CPI inflation.

As Table 2 reveals, we witness lags in all four crises. Even in deflationary periods like in the 1998–1999 Asian Financial Crisis and the 2001–2003 recession that followed, changes in median household income, and therefore the poverty line, fail to capture rising growing economic pressures on a population that is doubly suffering from higher unemployment rates. We observe as much with declines in the poverty line across all crises: from HK$9,000 to HK$8,700 in the Asian Financial Crisis, from HK$9,000 to HK$7,850 in the 2001–2003 recession, from HK$9,050 to HK$8,850 in the Financial Crisis, and most recently from HK$14,250 to HK$13,450 in the COVID-19 pandemic.

In other words, Eq. (3) lowers the bar for who counts as poor in times when it should least do so, namely, during times of economic crisis when a larger proportion of households are out of work and simultaneously paying more for necessities. It excludes a number of households that would have been considered poor in previous years, misleading us to believe the poverty rate is lower than it actually is when the poverty line is pegged to the median, rather than cost-of-living.

Let us now reassess the poverty line and poverty rate based on cost-of-living, rather than the median. Let us keep the same assumptions of Eq. (3), using households of 2.5 persons (*Q*_*g*_ = 2.5) as our basic unit of analysis for determining the number of goods to be consumed and benchmarking the poverty line, supposing that *t* intervals is one-year intervals, and treating the year 1996 is the base period (*t* = 0). With these assumptions in place, we can recalculate the cost of living with Eq. (5) using prices in nine categories from 1996 to 2020: food (including meat, fish, poultry, pork, bread, rice, fruit, vegetables, dairy products, drinks, sugar, flavorings, eating out), housing (including rent and property management company fees, as apartments are the most common type of residence in Hong Kong), clothing and footwear, durable goods (e.g., household appliances, furniture, etc.), miscellaneous goods (e.g., commodities that are non-durable, not clothing or footwear, and not food, such as jewellery, stationery, newspapers, etc.), transportation, miscellaneous services (e.g., health care, tutoring and education, banking, and other services). We thus arrive at *F*_*t=a*_ = 144.1 and a default of *F*_*t=0*_ = 100 that reflect a cost of living *L(X)* of HK$28,815.08 in 2020 compared to HK$20,476.20 in 1996.

**Fig. 5:**
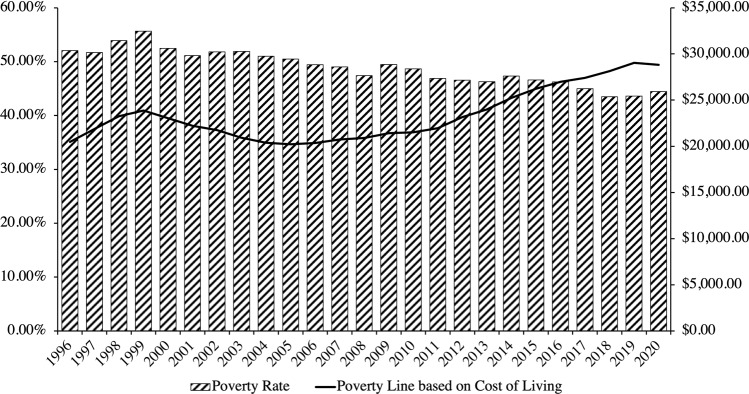
Poverty rate of Hong Kong based on Eq. (6) and cost-of-living from Eq. (5). Source: Author’s calculations based on data from the Census and Statistics Department of Hong Kong (2021b) and the Legislative Council of Hong Kong (2001).

Figure 5 traces the trajectory of the poverty rate recalculated with Eq. (6) based on the cost of living. Informative patterns emerge. Unlike Fig. 4, it is observed in Fig. 5 that the poverty rate has declined since 1996, rather than increased, *but* most salient is the distance in poverty rate and poverty line between the two charts—both appear in Fig. 5 to be multiples of what is reported in Fig. 3. Where in the standard relative measure of the poverty line in Fig. 3 sits at HK$13,450 in 2020 compared to HK$8,750 in 1996 (based on 50% of median household monthly income), producing a poverty rate of 23.6% in 2020 compared to 16.43% in 1996, the poverty line in Fig. 5 is determined to be HK$28,815 in 2020 compared to HK$20,476.20 in 1996, producing a poverty rate of 44.47% in 2020 compared to 52.07% in 1996.

**Fig. 6:**
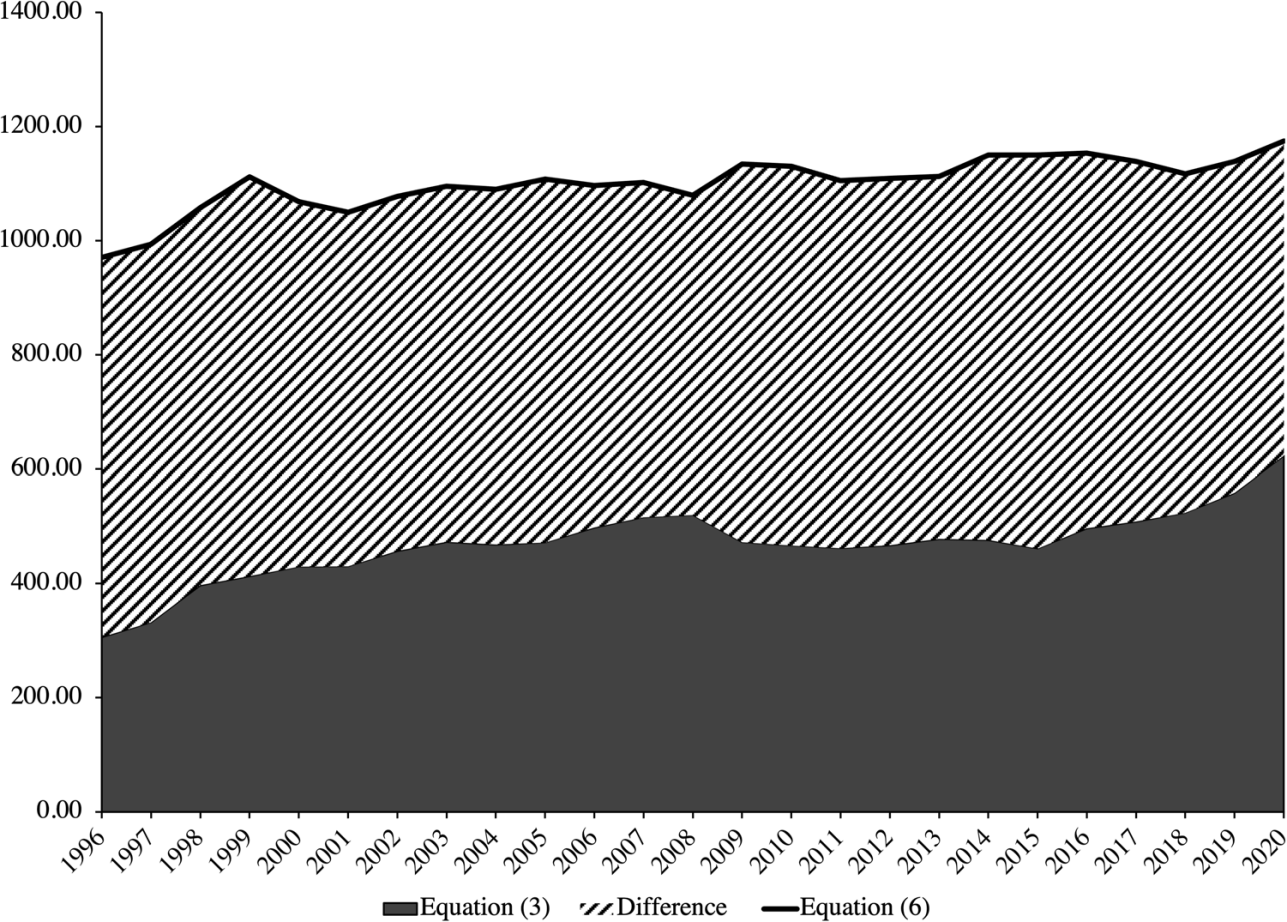
Number of households (in thousands) below the poverty line. Source: Author’s calculations based on data from the Census and Statistics Department of Hong Kong (2021b) and the Legislative Council of Hong Kong (2001).

Figure 6 expresses the rift in poverty rates in absolute terms based on the number of poor households according to the two equations. Based on Eq. (3), relative measures of poverty identify the number of households below poverty line to be about 623,540 in 2020. Based on Eq. (6), however, it is identified that the number of households below the poverty line is nearly double at 1,174,930. This discrepancy represents a shortfall of 551,400 poor households whose means are below the cost of living that have gone unaccounted for in determinations of the poverty rate in relative terms like a proportion of the median in Eq. (3).

## Discussion

In the measurement of inequality, a consensus holds to adopt the Gini Index as a scaled relative measure of economic distances within a population (Liao, 2021). Conventional relative measurements of the poverty rate are similarly based on the sum of individual income diversity or differences from the median income in a population (Brady, 2019).

Testing these two relative measures in Hong Kong, this article has demonstrated that they fail to capture changes in economic wellbeing and understate poverty. The standard Gini Index at an aggregate level (Eq. (1)) and configurations at group levels (Eq. (2)) fail to capture, and even mask, tectonic shifts in household income, and as a result, household size. This finding adds to Blesch et al. (2022) recent observation that the Gini Index is lacklustre as a single-parameter measure that fails to distinguish inequality concentrated at lower and top income percentiles, a point echoed in this article’s breakdown of changes in incomes by household size in Fig. 1. The breakdown of absolute income trends by household size illustrates the importance of what Osberg (2017) identifies as separating changes in income distribution and an index like the Gini that attempts to summarize this distribution.

In a similar vein, pegging the poverty to some proportion of the median as is convention (Eq. (3)) is demonstrated to lag behind shifts in economic means and cost-of-living, particularly during times of economic crisis. This lag is not unusual, given the non-linear relationship identified between long-run inflation and income (inequality), where lower and higher inflation rates are most destabilizing for income distributions by causing the largest rises in income inequality (Monnin, 2014). Though the mechanisms through which this effect takes shape are unclear, it bears remarking that the present context of Hong Kong has demonstrated real wage growth of 0.5% per year, a significant illustration not only of inflation, but of the vulnerability of households and their attendant poverty rate to economic crises (Au, 2023).

This article adopts a cost-of-living approach in the measurement of poverty. Calculating the poverty line based on long-term changes in the real costs of goods and services (Eq. (5)), this article demonstrates that relative measures of poverty, much like the Gini Index, underestimates the poverty rate by multiples, which translates into nearly half a million of poor households left unaccounted for. Measuring poverty in terms of cost-of-living (Eq. (6)), by contrast, captures these differences and expresses poverty more accurately as households whose economic means fall below cost-of-living at a given time in society, a more accurate depiction of poverty than an arbitrary level of income based on what someone else earns, which may fall while cost-of-living rises.

This article thus contributes to ongoing debates about the relative versus absolute measurement of poverty and inequality by demonstrating the merits of an intermediate approach based on cost-of-living. Absolute measures are argued to overlook the effects of income distribution on welfare (Brady, 2003), whereas relative measures are claimed to overlook absolute needs for nutritional wellbeing and survival (Sen, 1983, 2008).

A cost-of-living approach offers an alternative for this conundrum by determining a reference welfare level based on real costs, building an approach of basing the national poverty line on bundles of food requisite for nutritional survival in developing nations (Ravallion, 2012), yet extending it by encompassing a wider basket of essential goods and services as well as changes in their costs.

This approach gains credence from Ravallion’s (2020) observation that “existing poverty measures tend to opt for one of two very different assumptions, corresponding to the absolute and relative measures above: (a) that relative income does not matter to economic welfare or (b) that relative income is all that matters. Neither is plausible”. (p.168). The present approach echoes his skepticism in propounding the need for an alternative. But where Ravallion (Ravallion and Chen, 2019) moves on to develop an approach to account for both upward and downward relative comparisons in formulating a distribution-corrected mean income to benchmarking the poverty line, the present cost-of-living approach leans closer to Sen’s (1983) capabilities approach in accounting for material needs relevant to welfare.

## Limitations and future research

In calculating the cost-of-living in Hong Kong, this article borrows the same assumption that the city’s government used to calculate its aggregate poverty rate (20%) on a relative basis, namely, it pegs its inputs to the needs of a household equivalent to 2.5 persons. The limitations of this article thus similarly share in those espoused by the government measure: as the distribution of households across different sizes changes over time, a unit of analysis larger or smaller than 2.5 persons may be required, generating different inputs (and poverty rate) as a result. Indeed, as even Ravallion (2020) bemoans, “any price index found in practice has some implicit welfare anchor, and the index value will (in general) vary as the reference welfare level varies… national consumer price indices are typically anchored to consumption bundles somewhere around the mean or median of the distribution of income” (p.170). This study, in sum, offers but a cross-sectional view of poverty.

Similarly, the measurement of poverty using a cost-of-living approach partially accounts for the fact that certain needs are inelastic (like property management fees), which is an improvement above relative measures, but may not fare better than relative measures in not fully factoring in an important fact: the adjustments to purchases that households make once their income falls below the poverty line (and how to measure these adjustments) remain an area of uncertainty and fertile grounds for future research. As economists have long recognized, consumers have an elasticity of substitution of products within a module, such as replacing bread with rice, even if needs themselves do not change (Handbury, 2019).

Moreover, though this study is based on Hong Kong, an advanced capitalist economy in the Asia-Pacific region, it is an urbanized context where the overwhelming majority of workers work in service-oriented professions. Future studies might seek to identify inequality and discrepancies in the poverty rate in advanced capitalist economies in the Asia-Pacific region where the labor market has a larger share of agricultural or rural workers.

Finally, this article opens dialog on another area for future research: investigating the links between inequality and poverty themselves. In the present article, though the link was between the two was not investigated, it was implied that inequality serves as but a sensitizing device for the deeper issue of poverty that has eluded conventional relative measures. This is akin to Brady’s argument (2019), for instance, that states it is not evident whether “poverty is simply a subset of status attainment or if it can be explained by broader theories of the income distribution” (p.1).

On a meta-level, Korom (2019) observes through a bibliometric citation network analysis of inequality research in sociology and economics that scholars overwhelmingly tend to study wealth inequalities between ethnic groups—but strikingly reveals a differentiation between poverty research and research on wealth inequality as separate research domains or clusters. In other words, despite the theoretical common ground between poverty and inequality, they empirically, and therefore policy-wise, remain islands (DiPrete and Fox-Williams, 2021; Goubin, 2018).

## Data Availability

The data generated during and/or analyzed during the current study are available from the Census and Statistics Department of Hong Kong.
